# Hospital discharge planning in care transition of patients with chronic noncommunicable diseases

**DOI:** 10.1590/0034-7167-2022-0772

**Published:** 2023-12-04

**Authors:** Sara Maria Barbosa, Fabiana Costa Machado Zacharias, Tatiele Estefâni Schönholzer, Diene Monique Carlos, Maria Estela Lacerda Pires, Silvia Helena Valente, Luciana Aparecida Fabriz, Ione Carvalho Pinto

**Affiliations:** I Universidade de São Paulo. Ribeirão Preto, São Paulo, Brazil; II Universidade Federal do Paraná. Toledo, Paraná, Brazil; III Universidade Federal São Carlos. São Carlos, São Paulo, Brazil; IV Prefeitura Municipal de Batatais. Batatais, São Paulo, Brazil

**Keywords:** Transitional Care, Continuity of Patient Care, Process Assessment, Patient Discharge, Nurse’s Role, Patient-Centered Care., Cuidado de Transición, Continuidad de la Atención al Paciente, Alta del Paciente, Papel de la Enfermera, Atención Centrada en el Paciente., Cuidado Transicional, Continuidade da Assistência ao Paciente, Alta do Paciente, Papel do Profissional de Enfermagem, Assistência Centrada no Paciente

## Abstract

**Objective:**

to analyze care transition in hospital discharge planning for patients with chronic noncommunicable diseases.

**Method:**

a qualitative study, based on the Care Transitions Intervention theoretical model, with four pillars of intervention, to ensure a safe transition. Twelve professionals participated in a public hospital in the countryside of São Paulo. Data were collected through observation, document analysis and semi-structured interviews.

**Results:**

there was a commitment of a multidisciplinary team to comprehensive care and involvement of family members in patient care. The documents facilitated communication between professionals and/or levels of care. However, the lack of time to prepare for discharge can lead to fragmented care, impairing communication and jeopardizing a safe transition.

**Final considerations:**

they were shown to be important elements in discharge planning composition, aiming to ensure a safe care transition, team participation with nurses as main actors, early discharge planning and family involvement.

## RESUMO

**Objetivo:**

analisar a transição do cuidado no planejamento de alta hospitalar de pacientes com doenças crônicas não transmissíveis.

**Método:**

estudo qualitativo, fundamentado no modelo teórico *Care Transitions Intervention*, com quatro pilares de intervenção, para garantir uma transição segura. Participaram 12 profissionais, em hospital público, no interior paulista. Os dados foram coletados por meio de observação, análise documental e entrevistas semiestruturadas.

**Resultados:**

houve comprometimento da equipe multiprofissional para a integralidade da assistência e envolvimento de familiares nos cuidados ao paciente. Os documentos facilitaram a comunicação entre profissionais e/ou níveis de atenção. Todavia, a falta de tempo hábil para preparação da alta pode ocasionar um cuidado fragmentado, prejudicando a comunicação e colocando em risco a transição segura.

**Considerações finais:**

evidenciaram-se como elementos importantes na composição do planejamento de alta, visando garantir a transição do cuidado segura, participação da equipe com protagonismo do enfermeiro, planejamento precoce de alta e envolvimento da família.

## RESUMEN

**Objetivo:**

analizar la transición de la atención en la planificación del alta hospitalaria de pacientes con enfermedades crónicas no transmisibles.

**Método:**

estudio cualitativo, basado en el modelo teórico Care Transitions Intervention, con cuatro pilares de intervención, para garantizar una transición segura. Doce profesionales participaron en un hospital público del interior de São Paulo. Los datos fueron recolectados a través de observación, análisis de documentos y entrevistas semiestructuradas.

**Resultados:**

el equipo multidisciplinario se comprometió a brindar una atención integral e involucrar a los familiares en el cuidado del paciente. Los documentos facilitaron la comunicación entre profesionales y/o niveles de atención. Sin embargo, la falta de tiempo para prepararse para el alta puede dar lugar a una atención fragmentada, perjudicando la comunicación y poniendo en peligro la transición segura.

**Consideraciones finales:**

se mostraron elementos importantes en la composición de la planificación del alta, con el objetivo de garantizar una transición segura de la atención, la participación del equipo con los enfermeros como protagonistas, la planificación del alta temprana y la participación de la familia.

## INTRODUCTION

Chronic non-communicable diseases (NCDs), traditionally defined as long-term and slow-progressing diseases, are the main causes of death in the world and, therefore, one of the greatest health challenges today^(1)^. Complications arising from NCDs have consequences for individuals, for the health system and for society. Such illnesses lead to a decrease in quality of life, an increase in the cost to the health system, an increase in the demand for emergency care and hospitalizations, in addition to the occurrence of early retirement and costs to provide home care^(2-3)^.

Because they use different health services and have contact with different professionals, patients with NCDs may receive fragmented care, which significantly affects their health. As a result, there is a high demand for emergency services for patients with acute NCD problems^(4)^.

There is a group of innovative solutions that include discharge planning, seeking to improve integration, continuity of care and reduce frequent visits by users with chronic conditions to health services, which generates negative effects and rehospitalizations^(5-6)^. At hospital discharge, planning and health education for patients and their families is essential, especially in the care for patients with NCDs, in order to ensure continuity of care^(7)^.

The discussion about the importance of discharge planning, linked to user follow-up after the hospitalization period, gave rise, in the mid-1980s, to reflections on care transition (CT) and transitional care (TC)^(8-9)^. TCs are defined as a set of actions designed to ensure coordination and continuity of care as patients are transferred between different locations or at different levels of care^(10)^.

Actions for adequate CT are defined in the Care Transitions Intervention (CTI), a multidisciplinary CT program originally developed by Eric Coleman, University of Colorado, implemented in several hospitals in the same region. The program aims to facilitate the self-management of patients in their own health after hospitalization^(11)^.

There are four pillars or domains, which are considered essential for the development of this program: (1) medication self-management; (2) development of a personal health record, which is carried out from one location to another; (3) follow-up with follow-up in primary care; and (4) identification of “red flags” or signs of deterioration, which should lead these patients to contact the health service^(11)^. This is one of the best-known intervention models in the medical field.

This program consists of training patients and their families to become independent and confident when assuming their health condition. CTI interventions reduced early hospital readmission from 13.9% in the control group to 8.3% in the intervention group after 180 days. It also showed a significant reduction in the readmission rate, when compared to the control group (20%), with 12.8% in the intervention group^(12)^.

The absence of adequate discharge planning during this period of CT is recognized as a high-risk scenario for patient safety. Good planning aimed at transition safety has a direct impact on reducing hospital admissions and readmissions caused by complications^(10)^.

The negative results of the problems identified in this transition include: increased morbidity and mortality and adverse events; delays in receiving appropriate treatment; weak community support; higher frequency of visits to the emergency service; duplicate or missed exams during follow-up; avoidable hospital readmissions; emotional and physical pain, generating suffering for users, caregivers and/or family members; patient dissatisfaction with coordination and continuity of care^(13)^.

Given the above, the interest in this study arose from the analysis of domains or intervention pillars that guarantee a safe CT. To this end, the magnitude of the demands that chronic conditions generate for health systems were taken into account as well as the lack of articulation of this concept in the Brazilian context. CTI strategies have been highlighted as one of the ways to overcome the fragmentation of care and ensure continuity of care.

## OBJECTIVE

To analyze CT in hospital discharge planning for patients with NCDs.

## METHODS

### Ethical aspects

The research was developed in accordance with national and international ethics guidelines, being approved by the Research Ethics Committee of the *Escola de Enfermagem de Ribeirão Preto, Universidade de São Paulo* (EERP/USP), Certificate of Presentation for Ethical Consideration (CAAE - *Certificado de Apresentação para Apreciação Ética*), of March 24, 2021. The Informed Consent Form was obtained from all individuals involved in the study, in writing, personally.

### Study design

This is a holistic, exploratory, single case study with a qualitative approach, conducted and structured with reference to the Consolidated criteria for REporting Qualitative research (COREQ)^(14-15)^.

### Study setting

The study was carried out in a clinical inpatient unit of a medium-sized public hospital, a reference for 26 municipalities, located in the countryside of the state of São Paulo. The medical clinic sector has five semi-intensive beds, two isolation beds and 16 infirmary beds, to care for worsening clinical conditions of NCDs, with an average of 150 hospitalizations per month.

### Data source, collection and organization

For data collection, three complementary sources were used: documentary research, non-participant observation and semi-structured interviews.

In the document analysis, standardized forms and documents inserted in the hospital management software were photographed by the main researcher and transcribed into Word files, ensuring the structure of the documents as close as possible to their original format. Subsequently, they were analyzed.

Non-participant observation of the hospital discharge process was carried out by a doctoral nurse in July 2021. There was prior contact with the sector coordination to authorize the follow-up of all discharges that occurred in the period. The purpose of this step was to describe the discharge planning carried out for patients with NCDs and to identify tools (protocols, checklist, regulations, forms, routine and procedures) used by the health team in discharge planning. The entire discharge preparation process was observed, from the movement of professionals by the bed, to pass on the guidelines and deliver documents, to patients’ departure and the completion of discharge reports, through digital means.

During the data collection period, it was possible to monitor 20 (twenty) discharge processes, with an average duration of 20 (twenty) minutes each, making a total of 460 (four hundred and sixty) minutes of observation. The performance of all higher-level professionals, members of a multidisciplinary team, who participated in discharge planning, such as nurses, doctors, psychologists, occupational therapists and social workers, was observed. A field diary was prepared to record the information regarding observation. The discharge process was considered from the team’s daily meetings, to assess possible discharges, to the movement of professionals by patients’ bed to pass on the guidelines and delivery of documents, and completing discharge reports in the information system at the service desk.

In order to carry out the interviews, at first, a questionnaire was presented with data on participant characterization (age, length of experience, sex, professional category and education). Subsequently, a script was created with guiding questions based on the pillars of safe CTI. Respondents were health professionals who worked in the medical clinic sector who were willing to participate. Professionals who worked at the hospital for more than six months and developed activities in discharge planning were included. Professionals on vacation, on leave or on leave were excluded.

The study included 12 higher education professionals, who worked directly in the hospital discharge process. Only one clinical nurse was excluded, as he had been working in the medical clinic sector for only 4 months. Speech saturation occurred due to the repetition of themes in the tenth interview, however, it was decided to interview all professionals^(16)^. All professionals were personally invited and agreed to participate in the interviews. Each morning, the sector coordinator designated the professionals available that day, and the researcher was waiting for the best time to carry out the interviews in a room intended for the multidisciplinary team in the sector itself. The interviews lasted about 20 (twenty) minutes in July 2021. The interviews were identified with the letter I, corresponding to “interviewee”, followed by the numbers 1 to 12, sequentially.

The data resulting from the observation stage and document analysis contributed to the understanding of participants’ reports.

### Data analysis

Data were analyzed according to thematic analysis^(17)^, following a deductive logic guided by the four pillars for a safe transition^(11)^.

Document analysis enabled the identification of discharge reports, the main health information collected as well as the evolution records of each patient. Evaluative documents of patients’ clinical evolution and forecast of discharge are part of the scope of documents. With regard to the observation stage, the data were recorded in a field diary, which allowed a descriptive analysis of information.

The interviews were transcribed literally in a text editor and, in the sequence, the initial codes were made, then the grouping and/or separation phase. After this phase, the potential themes were transcribed onto a new page of the word text editor and grouped, in order to build a relationship between the data, to obtain, in the end, the central theme of the following thematic categories: *Follow-up with follow-up care primary; Medication self-management; Personal health record*; and *Identifying signs and symptoms of worsening condition*.

## RESULTS

In this study, respondents’ mean age was 28 years. As for job tenure in the sector, it was observed that the majority (n=8) had more than one year. As for gender, the majority (n=10) are female. Regarding the professional category, nurses, physicians, psychologists, occupational therapists and social workers were interviewed. It was found that nurses (n=8) were the most predominant professionals in terms of education, most (n=10) with a graduate degree.

The first pillar of CTI resulted in the category “Follow-up with follow-up in Primary Health Care”.

Considering the interviewees, continuity of care is guaranteed through comprehensive care in discharge planning, with the presence of a team working together, which ensures continuity of care and a safe transition, as it involves the family’s integrated participation in care during and after hospital discharge.

Professionals believe that family involvement in care, during preparation for discharge, should be carried out with a view to solving problems that are beyond the health points of that moment, in order to equip family members to continue care at home.

These aspects were identified in the records of documents analyzed in the observation, recurrent in interviewees’ speeches:

*I believe that when we bring the family together and start training here, the risk of patients coming back with the same problem is much lower. We empower family members here. Often, we are unable to do this for patients who go to a backup hospital. This involvement of the family and, later, with the network our RDH* [Regional Department of Health] *who is informed of what he will need. We believe that it guarantees the follow-up of care without having to return to the tertiary sector. (I3)*

*We approach the family, close to discharge, do training, check the needs and what patients will need for the basic network there for the primary sector. So, we forward our discharge plan of what we did and what they will need after discharge for them to continue care at home*. (I4)

*It’s how patients will continue their care at home, for example, if they will be able to continue with the medication that is already followed here at the hospital, follow the follow-up consultations in the return visits, see who will take care of them, the degree of care, the degree of dependency, see if what they need, where they live.* (I5)

To ensure comprehensive care implementation, it was possible to observe, in daily team meetings, the effective presence of multidisciplinary work in this sector, with emphasis on the evolution and discharge planning of each patient. During meetings, each professional presented patients’ discharge conditions and all aspects related to the transition to the next level of care. Participants seek consensus on the discharge of patients, which only happens if all professionals consider that there are adequate conditions for this.

That said, teamwork is perceived by respondents as an essential point in CT in the development of discharge planning:

*It is a process developed both with the medical team and with the nursing team and other areas to follow up on care after patients’ hospital discharge.* (I9)

*It depends on how the patients are, if the patient is bedridden, for example, the social worker already gets in touch with him where he lives in his municipality, who lives with them, their degree of dependence, what they will need for discharge. If they will need assistance to take medication, who will give it, who will help, all of this is seen before they leave, if they need oxygen, who will put it on, how they will put it on, how they will make it available, if they are able to receive it.* (I8)

Another important point to highlight, within discharge planning, is the early planning of this process. It was found, in document analysis, observation, and in interviewees’ speeches, that guidelines and planning are done before discharge and routinely in this institution:

*The discharge process starts with patients’ admission, we cannot think about discharge only when the medication ends or when a pathology is resolved.* (I5)

Only one of the interviewees pointed out the lack of time as a difficult factor for discharge planning:

*Here, turnover is high, it’s a very fast process, we don’t have the time we need to schedule a discharge. I feel bad about it. Kind of bad with myself for not being able to calmly look at a discharge, because you have to free up the bed, you have to do such a thing, you have to run. There was discharge, then on duty, let’s clean the bed and the next one will come [laughs]; it is difficult!* (I11)

It is also observed, in planning of care, that interviewed professionals are held accountable for ensuring the adequate counter-referral of this user:

*We have partnerships, we work a lot with health units and other hospitals and normally they have our phone number, our e-mail, if they need any support, we also give it to all the patients we discharged.* (I10)

Nurses’ role in CT and in the hospital discharge planning process was observed by the researcher and also pointed out in interviewees’ speech:

*I think what makes a discharge qualified is when we can talk to the medical team and the nursing team. I think that especially the nurse, he brings a lot of how this patient’s demand will be.* (I7)

Observation allowed us to state that nurses are one of the professionals most involved in discharge planning, for developing activities in care planning, assistance for social rehabilitation, health education, articulation with other health services and post-discharge follow-up to ensure continuity of care.

It is noted that nurses, in addition to taking the lead in discharge planning, also identify the need for other professionals to act to ensure complete assistance to users:

*Doctors can’t see all the planning. They just discharge. If you have oxygen, you leave with oxygen, but what then? At home? They don’t pay attention to these issues.* (I7)

Regarding the second pillar of intervention, self-management of medication, it was identified that there is guidance, on the part of nurses, on how patients will manage their medication reconciliation, inserting the new prescribed medications to those that were already used. In this regard, the statements indicate that nurses are responsible for this activity:

*Here, it’s the nurses themselves. Doctors show the prescription and say you are going to take this and this. We often have to ask doctors about medications. Sometimes it’s a 5pm discharge on a Friday, so we wonder if he’ll be able to get his medication from the health center. Does he have money to buy at the pharmacy? I don’t know! We try to release some medicine for him. Then the doctor has to prescribe, goes through the day hospital, through the RIN, but is nursing that does this.* (I5)

*Currently, nurses manage medication. I also ask residents to always talk, because many times patients will leave, for example, with an anticoagulant that he did not take before, you have to see which is the best anticoagulant that he will take. Often, SUS does not provide the best anticoagulant, so the family has to buy.* (I7)

The third pillar of intervention, personal health record, is an instrument in which the users themselves record their health condition, a type of diary, often used when patients undergo CT. However, it was not possible to identify, in the documents or during observation, this health tool in the investigated institution:

*As far as I know, there is no* [patient’s personal record]. *What we have is a satisfaction survey that goes to the email or SMS* [text message], *and he classifies, in fact, he writes about the hospital care, but he doesn’t talk about it, so this device that I I’ve never seen.* (I4)

The fourth pillar of intervention, identification and recognition of signs of users’ worsening condition, from health professionals’ perspective, is an important domain in CT of patients with NCDs. In the interviews, professionals brought the following statements regarding the guidelines for the signs of worsening:

*I usually advise, because most patients ask, what do I do if I get sick again? I call here, do I come? Am I going to the health center?* (I5)

## DISCUSSION

In order to improve CT, based on four pillars of intervention, to carry out safe and qualified transitions from hospital to home, the CTI was developed^(11)^. This program is based on an individualized care plan and the availability of healthcare professionals who understand patients’ goals and preferences^(18)^.

The results of this study, on follow-up with follow-up in primary care, point to elements such as comprehensive care for patients’ needs. Add to this is the holistic view of these individuals transcends a merely curative practice that considers individuals in their social and family context. In this context, Cecílio and Merhy^(19)^ state that comprehensive care for a patient in the hospital is the effort of a complete and holistic approach of each person with health needs who, for a certain period of their life, need hospital care.

However, what is still observed in health care in Brazil is a fragmented and disjointed practice, which results in segregation of individuals, without considering the context in which patients are inserted. Among the aspects that interfere with the guarantee of a full care model, there is a lack of resources, structure, political management and greater investments in professional training and health education^(20)^.

Comprehensive care constitutes a continuous challenge, due to the complexity of its operation. It involves different actors and articulation strategies, in different scenarios, which are configured in networks, whether in terms of care, management or construction of public policies^(21)^.

Family participation in hospital discharge planning was evidenced in this study. Respondents report that only when care is fully understood by patients, family members and/or caregivers, through daily training, is the possibility of discharge discussed. Family members should be a comprehensive part of any health intervention, at any stage of the disease and in all care contexts. The assumption points to the relevance of the relationship between care for individuals and their family context as an indispensable factor for comprehensive care. The family plays a significant role in the hospital, notably in the hospitalization of adults and older adults^(22)^.

The speeches indicate a point of high impact in the care of these patients, which is the work developed in the medical clinic sector by a multidisciplinary team, which results in broad and complete assistance for patients. This work was also identified during the researcher’s observation period, when verifying, in team meetings, constant dialogues regarding patient discharge planning.

The teamwork proposal has been conveyed as a strategy to face the intense process of specialization in the health area. This process tends to deepen, vertically, knowledge and intervention in individualized aspects of health needs^(23)^.

However, literature review studies point out that the specialized and biologicist clinical model is still characteristic of hospital settings. The aforementioned model affects the organization of work with a functionalist approach, not favoring creativity, professionals’ understanding about their own work and co-responsibility^(24)^.

It should also be noted that a factor that hinders this teamwork is often the high demand from users, linked to the insufficient number of personnel. In fact, these issues can have a negative impact on work development and operation, in addition to the low incentive of management for this practice^(25)^_._

A study carried out in southern Brazil proposes that hospital discharge planning can be carried out together with the multidisciplinary team, with the purpose of meeting all patients’ biological, psychological and social needs in an individualized and early manner^(26)^.

Based on the analysis of some of the findings of this research, in addition to the daily observations, it was verified that the counter-referral form was adequately completed, which seeks to reduce the existing fragmentation between health services and health professionals.

For the operationalization of health services, the functioning of the referral and counter-referral system is necessary, which refers to the mechanism for establishing communication. Through this system, it is possible to perceive, in health services, that users obtain continuity in the care offered, since each piece of information coming from different health professionals and different services is always valid for continuity of care for these individuals, seen as a whole and object of comprehensive care^(27)^.

Counter-referral, effected by completing the specific form, consolidates relevant patient information. With the feedback in hand, the unit that referred it can understand which behaviors were adopted in the unit to which it was referred, aiming at continuing the assistance in the unit of origin of users and promoting comprehensiveness^(21)^.

By observing the scenario under study, it was possible to identify nurses’ role, who are a reference in the team, to make contacts with other professionals, transfer patients, control documents, request transport and schedule exams. It is observed that nurses play an important role, being recognized as an essential element for the multidisciplinary team’s work.

According to Melo^(28)^, in a study on transfer of care, in Belo Horizonte, nurses were perceived as the most prominent actors during the transfer of responsibility for patients. The author considers nurses as the greatest articulator of care with other areas and professional categories, prioritizing each patient’s individualities.

The actions were confirmed by Lima *et al*.^(29)^, in an integrative review on CT activities, carried out by nurses, as they found a variety of activities in which nurses are involved. Among the activities, it is important to mention the education of patients to promote self-management of their health condition, construction of care protocols with proper participation and sharing of information by professionals and patients during transitions.

The second pillar that Coleman *et al*.^(11)^ highlight, in order to carry out a safe CT, is medication self-management. In this study, brief and poorly systematized interventions for medication self-management were identified. Although there is a multidisciplinary team active in care, it was found that, most of the time, nurses perform this activity to ensure the CT pillar in hospital discharge planning.

The increase in the incidence of NCDs is directly related to the demand for use of medication to control or delay the body’s deterioration process, which contributes to polypharmacy. Low medication compliance in this public is still a problem that affects between 50% and 60% of these patients^(30)^.

According to some studies, medication reconciliation programs can help to reduce medication-related problems. It is worth noting that medication conference activities and education on medication self-management can enhance the nursing team’s actions, favoring patient safety in CT^(31-32)^.

The third pillar of intervention is using a personal health record, which was not observed in this study, as an intervention strategy. The Personal Health Record (PHR) is a record in which data and other information related to patient care are preserved. This is in contrast to the widely used electronic medical records operated by institutions such as hospitals or health care facilities which contain data entered by physicians^(33)^. PHR’ intention is to provide a complete and accurate summary of an individual’s medical history in an accessible and online form. Health data in a PHR can include data from an outcome reported by a patient, results from a laboratory, among other devices.

The fourth pillar of intervention is related to training and knowledge of signs of worsening. In the interviewees’ reports, there was an interest in this strategy, but current hospital practice showed a reduced intervention, and not always present during discharge planning.

Research points out that in the literature there are few published works regarding nurses’ care activity in CT^(34)^. The importance of nurses’ role is highlighted, considering their capacity and ability to understand human beings comprehensively, meeting their needs and expectations, in order to make the individual and the family capable of emancipating and promoting care^(35)^.

The present study points to discharge planning as an essential tool for the multidisciplinary team, especially in patients with chronic conditions, even if not using all the pillars established by CTI. Another study, with a different transition protocol to CTI, was carried out with elderly North American patients, mostly female (67%) and non-Hispanic whites (84%). After intervention with a personalized care plan for patients and their families, it was observed that patients had a lower rate of return to the hospital within 30 days after discharge, and were more likely to attend medical appointments, in addition to being better prepared for CT^(36)^.

In a study that used other approaches, when considering users’ perception, it was also evidenced that discharge planning, through bonding and simple language, can collaborate in coordination of care and contribute to patients’ rehabilitation^(37)^.

### Study limitations

This study may present as a limitation the specificity of the qualitative and exploratory approach, in addition to the context of the study/medium-sized hospital, regional reference and linked to the university. Such aspects may hinder data transferability. Another limitation may be related to the predominant participation of female nursing professionals, which brings particularities to this professional category. Furthermore, it was not feasible for participants to check the final themes, an aspect that could bring new contributions. Considering the above, new studies are suggested with different approaches and different scenarios.

### Contributions to nursing, health, or public policies

It is expected that the results of this study provide subsidies for interventions to advance CT in hospital discharge in health services, in the sense of contributing to knowledge about discharge planning, an important activity when one wants to guarantee continuity and comprehensiveness of care as factors that can guarantee safe care.

## FINAL CONSIDERATIONS

The results of this study demonstrated that discharge planning, guided by strategies that aim to guarantee continuity of care comprehensively, guarantees the presence of an important pillar of intervention and monitoring with follow-up in Primary Care.

Health records, i.e., the documents standardized in the unit, were configured as a support for conveying information regarding patients’ health condition, and proved to be crucial for CT.

The family’s participation in care, during and after hospital discharge, and the presence of a multidisciplinary team are fundamental. Although nurses play a central role in this process and early discharge planning, the family and the team were highlighted as supporting this service, characterizing themselves as important elements that make up such activities.
